# Quantification of Mineralized Bone Response to Prostate Cancer by Noninvasive In Vivo μCT and Non-Destructive Ex Vivo μCT and DXA in a Mouse Model

**DOI:** 10.1371/journal.pone.0009854

**Published:** 2010-03-29

**Authors:** Murali Ravoori, Aneta J. Czaplinska, Charles Sikes, Lin Han, Evan M. Johnson, Wei Qiao, Chaan Ng, Dianna D. Cody, William A. Murphy, Kim-Anh Do, Nora M. Navone, Vikas Kundra

**Affiliations:** 1 Department of Experimental Diagnostic Imaging, The University of Texas M. D. Anderson Cancer Center, Houston, Texas, United States of America; 2 Department of Genitourinary Medical Oncology Research, The University of Texas M. D. Anderson Cancer Center, Houston, Texas, United States of America; 3 Department of Imaging Physics, The University of Texas M. D. Anderson Cancer Center, Houston, Texas, United States of America; 4 Department of Biostatistics, The University of Texas M. D. Anderson Cancer Center, Houston, Texas, United States of America; 5 Department of Radiology, The University of Texas M. D. Anderson Cancer Center, Houston, Texas, United States of America; National Cancer Institute, United States of America

## Abstract

**Background:**

To compare nondestructive in vivo and ex vivo micro-computed tomography (μCT) and ex vivo dual-energy-X-ray-absorptiometry (DXA) in characterizing mineralized cortical and trabecular bone response to prostate cancer involving the skeleton in a mouse model.

**Methodology/Principal Findings:**

In vivo μCT was performed before and 10 weeks after implantation of human prostate cancer cells (MDA-PCa-2b) or vehicle into SCID mouse femora. After resection, femora were imaged by nondestructive ex vivo specimen μCT at three voxel sizes (31 µ, 16 µ, 8 µ) and DXA, and then sectioned for histomorphometric analysis of mineralized bone. Bone mineral density (BMD), trabecular parameters (number, TbN; separation, TbSp; thickness, TbTh) and mineralized bone volume/total bone volume (BV/TV) were compared and correlated among imaging methods and histomorphometry. Statistical tests were considered significant if P<0.05. Ten weeks post inoculation, diaphyseal BMD increased in the femur with tumor compared to the opposite femur by all modalities (p<0.005, n = 11). Diaphyseal BMD by in vivo μCT correlated with ex vivo 31 and 16 µm μCT and histomorphometry BV/TV (r = 0.91–0.94, P<0.001, n = 11). DXA BMD correlated less with bone histomorphometry (r = 0.73, P<0.001, n = 11) and DXA did not distinguish trabeculae from cortex. By in vivo and ex vivo μCT, trabecular BMD decreased (P<0.05, n = 11) as opposed to the cortex. Unlike BMD, trabecular morphologic parameters were threshold-dependent and when using “fixed-optimal-thresholds,” all except TbTh demonstrated trabecular loss with tumor and correlated with histomorphometry (r = 0.73–0.90, P<0.05, n = 11).

**Conclusions/Significance:**

Prostate cancer involving the skeleton can elicit a host bone response that differentially affects the cortex compared to trabeculae and that can be quantified noninvasively in vivo and nondestructively ex vivo.

## Introduction

Prostate carcinoma is the most frequently diagnosed visceral cancer and the second most common cause of cancer-related death among American men [1], [2]. It has a proclivity to metastasize to bone [3], [4]; and, this is believed to be due not only to passive hemodynamic factors, but also the bone marrow microenvironment [5]–[7]. There is interplay between the tumor and the host bone, with each affecting the other. Because the prognosis for patients with prostate cancer deteriorates markedly once the disease escapes the gland, greater understanding of the interaction of prostate cancer with bone is needed. However, progress in such research has been limited by the need for nondestructive, quantitative imaging methods.

Because of the low levels of bone metastasis with prostate cancer models [8]–[15], techniques for direct injection into bone have been established [16]–[19]. Using such a mouse model, we have previously demonstrated that prostate cancer burden involving bone can be quantified using MR [20]. The ability to nondestructively image mineralized bone at high resolution under controlled experimental conditions in animal models [21], [22] is also needed. For small animals, micro-CT (μCT), which has spatial resolution in the order of microns, can be utilized. With the advent of newer generation clinical CT scanners, spatial and temporal resolution have continuously improved, suggesting assessment of trabeculae will soon be feasible with clinical scanners. Thus, knowledge of critical parameters for such assessment is needed.

Dual-energy X-ray absorptiometry (DXA) is commonly used clinically to measure bone mineral density BMD [23], [24]. However, 2D DXA provides relatively low spatial resolution compared to μCT and does not distinguish trabeculae. Bone histomorphometry can be used to assess the trabecular structure in animals, but it is invasive, destructive, as well as labor and time intensive.

μCT provides three-dimensional (3D) representations of bones and is now available at various resolutions [25]. However, the affect of thresholding on gross measures such as BMD and morphologic trabecular parameters is as yet unclear. Most μCT work has used automated thresholding techniques that normalize to bone density within the region of interest, but the density may be altered in pathologic conditions such as osteopenia, and more so with localized disease such as prostate cancer. We hypothesized that fixed “optimal thresholds” would be superior to commonly used “automated thresholds,” which vary by each object examined, in a quantifying trabecular response to prostate cancer. With localized disease, we hypothesized that the affect of prostate tumor on trabecular-mineralized bone may vary from mineralized cortical bone since cancer generally first involves the marrow. Such evaluation may be afforded by the three dimensional nature of μCT. Since μCT can also be performed non-invasively in vivo, it is desirable because it enables longitudinal studies of the response of mineralized bone to prostate cancer; however, it commonly provides less spatial resolution than ex vivo μCT; therefore, comparison with ex vivo analyses is needed.

We compared the ability of in vivo μCT, ex vivo specimen μCT, and ex vivo DXA to characterize mineralized cortex and trabeculae in a mouse model of prostate cancer involving bone.

## Materials and Methods

### 1.1. Cell Culture

MDA-PCa-2b human prostate cancer cells were grown [20] in BRFF-HPC1 medium (Biological Research Faculty and Facility, Athena Environmental Services, Baltimore, MD, USA) supplemented with 20% fetal bovine serum (Life Technologies, Inc., Gaithersburg, MD, USA), 91 U/ml penicillin, 91 µg/ml streptomycin, and 2 mM glutamine. MDA-PCa-2b cells have been shown to engraft in the marrow cavity of bones, to be hormonally responsive, and to produce prostate-specific antigen [26]–[29].

### 1.2. Animals

Eight-week-old male severe combined immunodeficient (SCID) mice (n = 14) were purchased from Charles River Laboratories (Wilmington, MA, USA) and housed in specific-pathogen-free conditions. They were cared for in accordance with guidelines set forth by the Association for Assessment and Accreditation of Laboratory Animal Care and the U.S. Public Health Service Policy on Humane Care and Use of Laboratory Animals. The University of Texas M. D. Anderson Cancer Center's Institutional Animal Care and Use Committee approved all studies.

### 1.3. In Vivo Studies

For in vivo imaging procedures, SCID mice were anesthetized by inhalation of 2% isoflurane. The femora (n = 14 mice) were first scanned using in vivo μCT as described below. After baseline imaging, 5×10^5^ MDA-PCa-2b cells [26], [29] in 5 µl of growth medium were injected into the distal epiphysis of the right femur of each mouse [26], [29]. The distal epiphysis of the contralateral femur was injected with 5 µl of vehicle (Phosphate Buffer Solution (PBS)) to serve as a control. Three mice died immediately after tumor injection.

Ten weeks after tumor inoculation, the femora were again imaged in vivo by μCT (n = 11). The animals were euthanized and the femora were removed. The disarticulated femora without muscle were fixed in formalin and stored in 10% ethanol for ex vivo specimen μCT and ex vivo DXA. The femora were then processed for bone histomorphometry.

### 1.4. In Vivo μCT

For in vivo scans, SCID mice were imaged supine using a μCT scanner at a 91-µm isotropic voxel size (model RS-9, General Electric Medical, London, Ontario, Canada). The scanner has a fixed tungsten anode with a focal spot size of 50×30 µm. Images of the femora were acquired at an isotropic voxel size of 91×91×91 µm using the following scan parameters: 80 kVp, 450 µA, 100 msec per frame, and 3 frames per view.

A calibration standard was positioned in the field-of-scan view to enable the conversion of Hounsfield units (HU) into BMD values. MicroView software (version: 2.1.1; General Electric Medical) was used to view the images and calculate the BMD (in mg/cm^3^) of each femoral diaphysis. For this calculation, a standard measuring cylinder (2.5×2.5×5 mm) was placed so that its bottom was 1.5 mm above the bone's growth plate. BMD was calculated for the right and left femora, and the absolute difference in BMD values between the right and left femora was determined. Another cylinder (1.5×1.5×0.6 mm) was centered in all three planes within the metaphysis (excluding cortical bone) with the bottom at the beginning of the growth plate for the measurement of BMD and trabecular morphometric parameters. These parameters (TbN, number of trabeculae/mm; TbSp, trabecular separation/mm; TbTh, trabecular thickness/mm; and BV/TV, mineralized bone volume per total bone volume/%) were measured at fixed thresholds and at automatic thresholds (with values determined by the software) of mineralized bone. The BMD, TbTh, TbN, TbSp, and BV/TV obtained by μCT were correlated with the values obtained by bone histomorphometry. “Optimal threshold” was defined as the threshold that resulted in maximum correlation between the trabecular parameter by μCT and that by bone histomorphometry.

### 1.5. Ex Vivo specimen μCT

Ex vivo specimen μCT of the same femora was performed using 31-µm, 16-µm, and 8-µm voxel sizes on an Explore Locus SP preclinical specimen scanner (General Electric Medical). This scanner has a cone-beam volume CT system that uses a tungsten source X-ray tube operating at 80 kV and 80 µA. The object in the scanner is rotated in 0.4-degree increments on a holder between the X-ray source and charge-coupled device-based detector. Each bone specimen required approximately 4 hours for data acquisition. After raw images were normalized and defective detector pixels were corrected, a low-resolution scout volume was reconstructed using a modification of the method used by Feldkamp et al. [25] The scout volume was used to select the coordinates for a distal femur volume of interest, which was reconstructed into 31-µm, 16-µm, and 8-µm isotropic voxels. The images were viewed and analyzed for BMD, TbTh, TbN, TbSp, and BV/TV using the MicroView program as described above. We also obtained maximum gray-scale fixed-threshold values for both the diaphysis and metaphysis of each femur. Because of its limited field of view, the 8-µm acquisition covered only the metaphysis.

### 1.6. DXA

Matched left and right femur pairs from the 11 SCID mice were scanned ex vivo in a sagittal plane using a DXA scanner (Norland Medical Systems, Inc., New York, NY USA). A rectangular (2.5×5 mm) region of interest that encompassed the diaphysis was placed over each femur to obtain the BMD (in g/cm^2^). Because the trabeculae were not distinguishable from cortical bone, trabeculae were not analyzed with DXA.

### 1.7. Bone Histomorphometry

Representative 5-µm-thick sagittal sections through the entire width of the femur were obtained at three different levels (right, mid, and left) for bone histomorphometry measurements. The three histologic sections per femur were analyzed after Von Kossa staining for mineralized bone [25]. Cortical thickness was visually assessed and compared with visual assessments of μCT, DXA, and histomorphometry data. Using a trabecular analysis system (Osteometrics, TAS, version 20.8, Atlanta, GA, USA), the images obtained from the sagittal sections were viewed, and a rectangular region of interest was placed over each femur diaphysis (2.5×5 mm) or metaphysis (1.5×0.6 mm) in order to measure TbTh, TbN, TbSp, and BV/TV. Mineralized bone was calculated in terms of BV/TV, as described above. Care was taken to match the size and placement of the rectangular region of interest with the size and placement of the cylindrical region used in the MicroView program to evaluate the μCT data sets.

### 1.8. Statistical Analysis

Linear regression was performed to analyze correlations between values obtained from in vivo and ex vivo μCT, DXA, and bone histomorphometry studies using Excel software (2003 SP2; Microsoft, Bellevue, WA, USA). Student's t-test (two-sided) was used to compare values between the right and left femora. Pearson's correlation coefficient was calculated for differences between imaging techniques, and Fisher's Z transformation of the correlation coefficient was employed using SAS software (SAS Institute, Cary, NC, USA). For all tests, P<0.05 was considered significant.

## Results

### 2.1. Change in Diaphyseal BMD with Prostate Cancer Involvement

**Figure 1:**
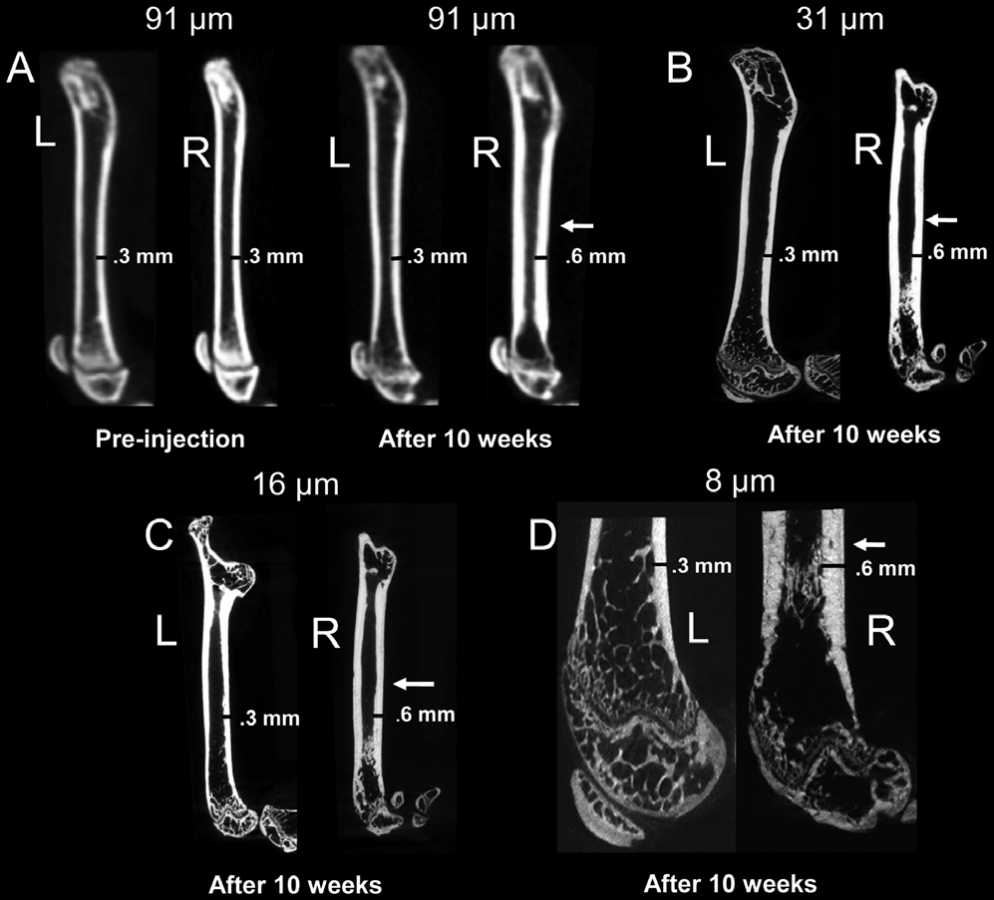
Representative in vivo and ex vivo μCT images demonstrate cortical thickening induced by prostate cancer involving bone. Sagittal in vivo longitudinal μCT scans obtained at 91 µm voxel size before and 10 weeks after (A) intrafemoral injection of vehicle control (left images) or MDA-PCa-2b human prostate cancer cells (right images) show cortical thickening (arrow) in the femora containing tumors. Ex vivo specimen μCT scans of excised femora at voxel sizes of 31-µm (B), 16-µm (C), and 8-µm (D) also show cortical thickening of the right femur with tumor. At the 8-µm voxel size, only the distal aspect of the femur was covered by the scans.

**Figure 2:**
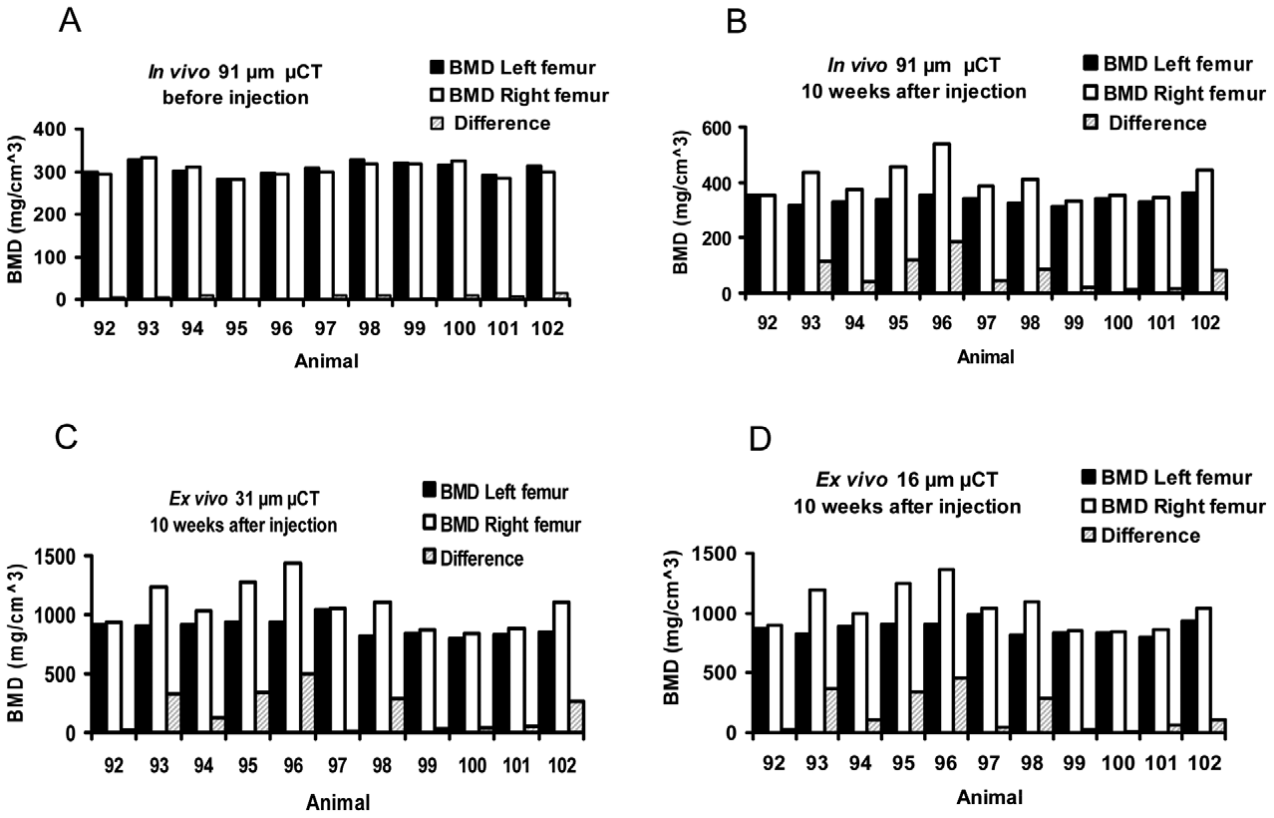
Bone mineral density (BMD) of the diaphysis was greater in the femora involved by prostate cancer. Diaphyseal BMD was measured in both femora by in vivo μCT before injection (A) and 10 weeks after injection (B) of vehicle (left femora) or prostate cancer cells (right femora), and then by ex vivo specimen μCT at 31 µm (C) and 16 µm (D) voxel sizes.

**Figure 3:**
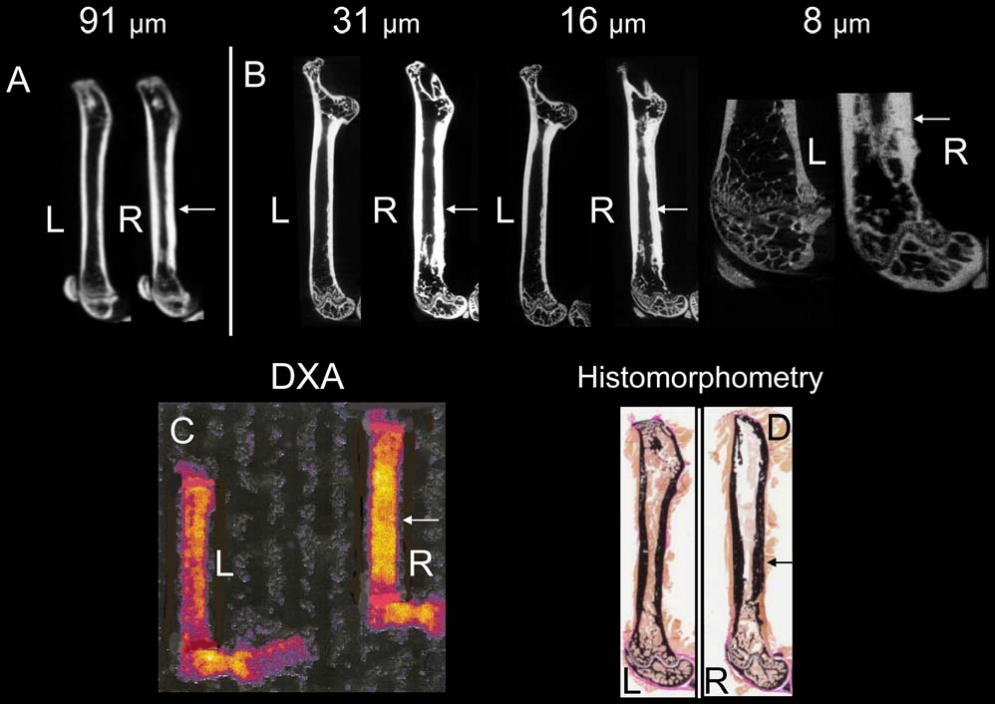
Representative in vivo and ex vivo images demonstrate morphologic changes of mineralized cortical and trabecular bone induced by prostate cancer involving the skeleton. Sagittal in vivo μCT and ex vivo specimen μCT images of femora 10 weeks after injection of vehicle (left) or prostate cancer cells (right). (A) In vivo μCT (91-µm voxel size); (B) ex vivo specimen μCT at 31-µm, 16-µm, and 8-µm voxel sizes; (C) ex vivo dual-energy X-ray absorptiometry (DXA); and (D) bone histomorphometry images are presented. L, left femur; R, right femur with tumor; arrow, cortical thickening.

Qualitatively, in vivo μCT showed no apparent difference in cortical thickness between the left and right femora before tumor injection; whereas, diaphyseal cortical thickening and trabecular alterations were noted using in vivo and ex vivo μCT 10 weeks after inoculation of the right femur with prostate cancer cells, but not the control left femur injected with vehicle (**Figure 1**). Quantitatively (**Figure 2**), there was no difference in BMD between the left and right femoral diaphyses before tumor injection by in vivo μCT. The mean absolute difference (MAD) in BMD between right and left femoral diaphyses was 6.9±4.3 mg/cm^3^. Ten weeks after tumor inoculation, the BMD of the femora with tumors increased (P<0.003), with a MAD of 65.91±57.6 mg/cm^3^. Results were concordant between in vivo μCT and ex vivo specimen μCT with voxel sizes of 31-µm (P<0.005, n = 11, MAD = 182.9±168.7 mg/cm^3^) and 16 µm (P<0.005, n = 11, MAD = 167.5±163.4 mg/cm^3^). The variations in standard deviations are due to biologic differences in tumor growth and host response in individual animals, yet significant differences (P<0.01) were noted. Representative images show that all methods (in vivo μCT at 91 µm voxel size, ex vivo specimen μCT at 31, 16, and 8-µm voxel sizes, DXA, and bone histomorphometry) demonstrated host bone response to the prostate cancer (**Figure 3**), including cortical thickening of the diaphysis (P<0.01, n = 11, by 91, 31, and 16-µm voxel size CT, DXA, and bone histomorphometry, **Figure 2** and data not shown). However, DXA did not distinguish the trabecular change, whereas, μCT and bone histomorphometry did.

### 2.2. Correlation of Diaphyseal BMD Values Obtained by In Vivo and Ex Vivo μCT, Ex Vivo DXA, and Bone Histomorphometry

**Figure 4:**
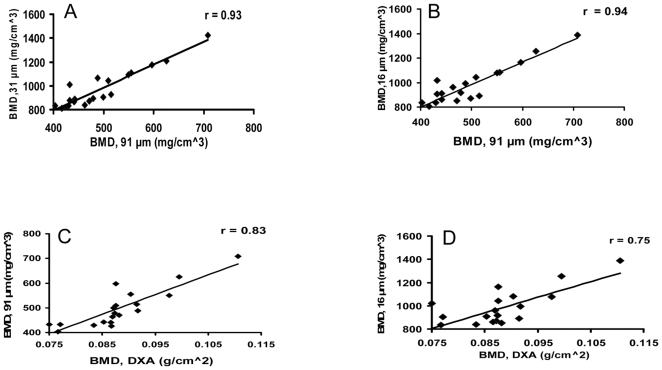
In vivo μCT and ex vivo specimen μCT measurements of bone mineral density (BMD) correlate with each other and with ex vivo dual-energy X-ray absorptiometry (DXA) BMD measurements. (A) In vivo μCT at 91-µm vs ex vivo μCT at 31-µm voxel size. (B) In vivo μCT at 91-µm vs ex vivo μCT at 16-µm voxel size. (C) In vivo μCT at 91-µm vs ex vivo DXA. (D) Ex vivo μCT at 16-µm vs ex vivo DXA.

There was high correlation of diaphyseal in vivo μCT BMD and ex vivo μCT BMD and BMD among ex vivo μCT at 31-µm- and 16-µm-voxel size (**Figure 4** **,** **Table 1**). In comparison, BMD correlated moderately between DXA and in vivo μCT, or ex vivo μCT (**Figure 4** **,** **Table 1**).

**Table 1:** Correlations between diaphyseal or metaphyseal BMD determined by bone histomorphometry and *in vivo* μCT, *ex vivo* specimen μCT or *ex vivo* DXA 10 weeks after tumor inoculation.

Technique	BMD At diaphysis		BMD At metaphysis	
	r	P	r	P
91 µm vs DXA	0.83	<0.001	-	-
31 µm vs DXA	0.73	<0.001	-	-
16 µm va DXA	0.75	<0.001	-	-
91 µm vs 31 µm	0.93	<0.001	0.95	<0.001
91 µm vs 16 µm	0.94	<0.001	0.95	<0.001
91 µm vs 8 µm	-	-	0.97	<0.001
31 µm vs 16 µm	0.98	<0.001	0.96	<0.001
31 µm vs 8 µm	-	-	0.95	<0.001
16 µm vs 8 µm	-	-	0.97	<0.001
91 µm vsHistomorphometry	0.92	<0.001	0.78	<0.001
31 µm vsHistomorphometry	0.91	<0.001	0.75	<0.001
16 µm vsHistomorphometry	0.92	<0.001	0.77	<0.001
8 µm vsHistomorphometry	-	-	0.84	<0.001
DXA vsHistomorphometry	0.73	<0.001	-	-

*In vivo μCT, 91 µm; Ex vivo μCT, 31, 16, 8 µm.

**Figure 5:**
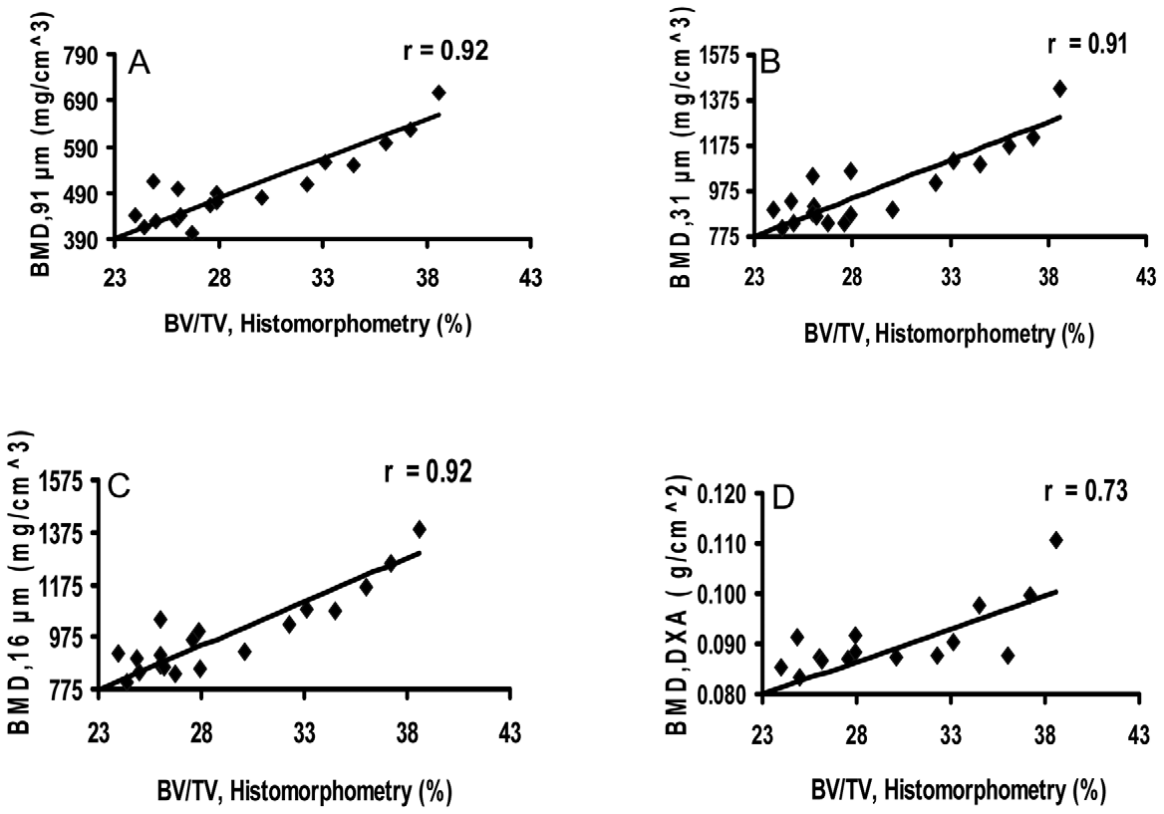
In vivo μCT, ex vivo specimen μCT, and DXA measurements of diaphyseal bone mineral density (BMD) correlate with histomorphometry. In vivo μCT (91-µm, A), ex vivo specimen μCT (31-µm, B; 16-µm, C), and DXA (D) measurements of bone mineral density (BMD) correlate with histomorphometric measurements of mineralized bone volume/total bone volume (BV/TV).

Diaphyseal BMD by in vivo μCT or ex vivo μCT at 31-µm- or 16-µm-voxel size correlated highly with BV/TV by bone histomorphometry (**Figure 5** **,** **Table 1**). In comparison, DXA BMD correlated moderately with the bone histomorphometry BV/TV (**Figure 5** **,** **Table 1**). In addition, the correlations between in vivo μCT and ex vivo μCT, at 31-µm or 16-µm, were higher than those between DXA and ex vivo μCT, at 31-µm or 16-µm (**Table 1**).

### 2.3. Metaphyseal Parameters and Fixed Threshold

**Figure 6:**
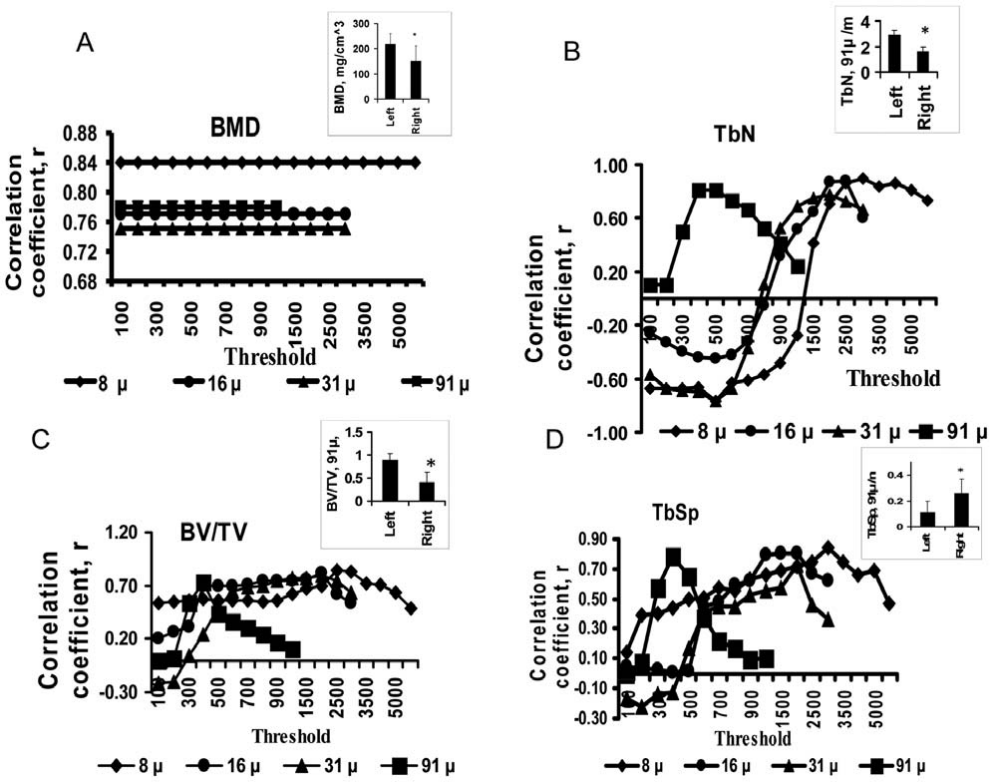
Assessment of trabecular morphologic parameters is threshold dependent; and, trabecular parameters are altered by prostate cancer involving bone. Variations in correlation coefficient (r) for metaphyseal bone mineral density (BMD, A), trabecular number (TbN, B) bone volume density (BV/TV, C), and trabecular separation (TbSp, D) by μCT at different thresholds and bone histomorphometry. Insets: using optimal thresholds, in vivo μCT at 91-µm voxel size shows significant differences in metaphyseal BMD (BMD, A), TbN (TbN, B), BV/TV (BV/TV, C) and TbSp (TbSp, D) between left and right femora 10 weeks after tumor inoculation.

Because trabeculae were located primarily in the metaphysis, we focused on the metaphysis for trabecular assessments. We compared trabecular BMD and morphometric parameters obtained by in vivo or ex vivo μCT at different thresholds with bone histomorphometry. Thresholding did not affect trabecular BMD obtained using in vivo or ex vivo μCT, but did affect μCT-derived morphometric parameters such as TbN, BV/TV, TbSp (**Figure 6, Tables 2 and 3**), and TbTh (**Tables 2 and 3**). μCT performed at each voxel size had individual narrow ranges for best thresholds for maximum correlation of trabecular parameters when compared to bone histomorphometry and these narrow ranges overlapped for TbN, BV/TV, and TbSp. At all voxel sizes and thresholds, μCT-derived TbTh did not correlate well with TbTh by bone histomorphometry (r<0.17, P>0.47, n = 11), likely because the small size of trabeculae caused volume-averaging artifacts.

**Table 2:** Correlations between metaphyseal BMD and bone structural parameters derived by bone histomorphometry, in vivo μCT, or ex vivo specimen μCT 10 weeks after tumor inoculation.

Technique and parameter	Model	Threshold (HU)	r	P
*In vivo* μCT, 91 µm vs Histomorphometry				
TbN	O*	400	0.81	<0.001
	A*	633±220	0.53	<0.02
TbSp	O	400	0.78	<0.001
	A	633±220	0.60	<0.002
BV/TV	O	400	0.73	<0.001
	A	633±220	0.58	<0.003
BMD	O	400	0.78	<0.001
	A	633±220	0.78	<0.001
*Ex vivo* μCT, 31 µm vs Histomorphometry				
Tb N	O	2000	0.78	<0.001
	A	1830±650	0.61	<0.002
Tb Sp	O	2000	0.73	<0.001
	A	1830±650	0.57	<0.001
BV/TV	O	2000	0.79	<0.001
	A	1830±650	0.58	<0.001
BMD	O	2000	0.75	<0.001
	A	1830±650	0.75	<0.001
*Ex vivo* μCT, 16 µm vs Histomorphometry				
Tb N	O	2000	0.88	<0.02
	A	2423±770	0.73	<0.3
Tb Sp	O	2000	0.8	<0.001
	A	2423±770	0.61	<0.001
BV/TV	O	2000	0.82	<0.001
	A	2423±770	0.69	<0.001
BMD	O	2000	0.77	<0.001
	A	2423±770	0.77	<0.001
*Ex vivo* μCT, 8 µm vs Histomorphometry				
TbN	O	3000	0.90	<0.001
	A	1822±920	0.71	<0.001
TbSp	O	3000	0.84	<0.001
	A	1822±920	0.69	<0.001
BV/TV	O	3000	0.83	<0.001
	A	1822±920	0.71	<0.001
BMD	O	3000	0.84	<0.001
	A	1822±920	0.83	<0.001

*O, optimal threshold; A, automatic threshold.

**Table 3:** Metaphyseal bone structural parameters determined by in vivo μCT, ex vivo specimen μCT, and bone histomorphometry 10 weeks after tumor inoculation.

Technique and parameter	Model	Threshold	Mean Value		
			Femur w/o tumor	Femur with tumor	P
*In vivo* μCT, 91 µm					
TbN (/mm)	O*	400	2.9	1.6	<0.05
	A*	633±220	2.4	2.2	<0.50
TbSp (mm)	O	400	0.11	0.26	<0.002
	A	633±220	0.41	0.54	<0.50
BV/TV (%)	O	400	0.89	0.4	<0.002
	A	633±220	0.25	0.16	<0.08
TbTh (mm)	O	400	0.3	0.13	<0.001
	A	633±220	0.13	0.11	<0.13
BMD (mg/cm^3^)	O	400	217	150	<0.004
	A	633±220	227	150	<0.004
*Ex vivo* μCT, 31 µm					
TbN (/mm)	O	2000	2.88	1.2	<0.005
	A	1830±650	2.95	2.24	<0.12
TbSp (mm)	O	2000	0.5	9.96	<0.05
	A	1830±650	0.32	0.45	<0.48
BV/TV (%)	O	2000	0.11	0.05	<0.02
	A	1830±650	0.12	0.11	<0.78
TbTh (mm)	O	2000	0.039	0.034	<0.15
	A	1830±650	0.041	0.054	<0.03
BMD (mg/cm^3^)	O	2000	451	342	<0.02
	A	1830±650	463	342	<0.02
*Ex vivo* μCT, 16 µm					
TbN (/mm)	O	2000	4.2	1.77	<0.001
	A	2423±770	3.4	2.14	<0.03
TbSp (mm)	O	2000	0.29	1.08	<0.02
	A	2423±770	0.3	0.54	<0.20
BV/TV (%)	O	2000	0.13	0.05	<0.005
	A	2423±770	0.1	0.06	<0.03
TbTh (mm)	O	2000	0.032	0.035	<0.56
	A	2423±770	0.03	0.034	<0.20
BMD (mg/cm3)	O	2000	475	344	<0.006
	A	2423±770	482	353	<0.006
*Ex vivo* μCT, 8 µm					
TbN (/mm)	O	3000	5.7	2.4	<0.001
	A	1822±920	13	37	<0.001
TbSp (mm)	O	3000	0.25	2.17	<0.05
	A	1822±920	0.1	0.05	<0.06
BV/TV (%)	O	3000	0.1	0.04	<0.02
	A	1822±920	0.16	0.31	<0.03
Tb Th (mm)	O	3000	0.02	0.016	<0.40
	A	1822±920	0.015	0.013	<0.39
BMD (mg/cm^3^)	O	3000	500	333	<0.006
	A	1822±920	486	339	<0.006
Histomorphometry					
TbN (/mm)			3.96	2.45	<0.003
TbSp (mm)			0.22	0.29	<0.05
BV/TV (%)			10	6	<0.006
TbTh (mm)			0.036	0.039	<0.27

*O, optimal threshold; A, automated threshold.

The automatic threshold or maximum gray-scale threshold usually did not return the optimal threshold (**Table 2**). The automatic threshold ranged from 550 to 975 HU in the left femora and 350 to 1250 HU in the right femora, and the values varied between animals and voxel sizes. Similarly, the maximum gray-scale threshold values ranged from 706 to 855 HU in the left femora and 744 to 915 HU in the right femora, and these values also varied between animals and voxel sizes. Correlations between μCT and histomorphometric TbN, BV/TV, and TbSp values were higher using fixed optimal thresholds than using automatic thresholds (**Table 2**). Trabecular parameters (TbN, BV/TV & TbSp) derived using optimal thresholds correlated among in vivo and ex vivo μCT (**Table 4**), and among in vivo or ex vivo μCT and histomorphometry (**Table 2**). Although we did not find good correlations between TbTh values obtained by μCT and bone histomorphometry, TbTh correlated moderately between in vivo and ex vivo μCT at 16, 31, or 8-µm voxel sizes, and among ex vivo μCT at the three voxel sizes (**Table 4**). Therefore, we used fixed optimal thresholds at different voxel sizes to compare trabecular parameters on μCT with those on bone histomorphometry.

**Table 4:** Correlations between metaphyseal morphometric parameters (TbN, TbSp, BV/TV, and TbTh) determined by in vivo μCT vs ex vivo specimen μCT at optimal thresholds.

Parameter and μCT technique	r	P
TbN, 91 µm vs 31 µm	0.78	<0.001
TbN, 91 µm vs 16 µm	0.81	<0.001
TbN, 91 µm vs 8 µm	0.76	<0.001
TbN, 31 µm vs 16 µm	0.88	<0.001
TbN, 31 µm vs 8 µm	0.85	<0.001
TbN, 16 µm vs 8 µm	0.89	<0.001
TbSp, 91 µm vs 31 µm	0.77	<0.001
TbSp, 91 µm vs 16 µm	0.77	<0.001
TbSp, 91 µm vs 8 µm	0.88	<0.001
TbSp, 31 µm vs 16 µm	0.81	<0.001
TbSp, 31 µm vs 8 µm	0.84	<0.001
TbSp, 16 µm vs 8 µm	0.74	<0.001
BV/TV, 91 µm vs 31 µm	0.88	<0.001
BV/TV, 91 µm vs 16 µm	0.86	<0.001
BV/TV, 91 µm vs 8 µm	0.74	<0.001
BV/TV, 31 µm vs 16 µm	0.87	<0.001
BV/TV, 31 µm vs 8 µm	0.79	<0.001
BV/TV,16 µm vs 8 µm	0.82	<0.001
TbTh, 91 µm vs 31 µm	0.43	<0.05
TbTh, 91 µm vs 16 µm	0.24	<0.27
TbTh, 91 µm vs 8 µm	0.58	<0.005
TbTh, 31 µm vs 16 µm	0.46	<0.03
TbTh, 31 µm vs 8 µm	0.69	<0.001
TbTh, 16 µm vs 8 µm	0.6	<0.003

*In vivo μCT, 91 µm; Ex vivo μCT, 31, 16, 8 µm.

### 2.4. BMD of Trabeculae of Metaphysis

In vivo and ex vivo μCT demonstrated decreases in the BMD of the metaphyseal trabeculae in femora with tumors compared to control femora at all voxel sizes (P<0.05, n = 11, **Figure 6A, Table 3**). The BMD of the trabeculae at the metaphyses was highly correlated between in vivo μCT and ex vivo μCT at all voxel sizes (**Table 1**).

### 2.5. Morphometric Trabecular Parameters

Trabeculae appeared better defined with decreasing voxel size of μCT, and trabecular thinning, thickening, and loss could be identified **(** **Figures 1** **and** **3** **)**. With automatic or optimized thresholds, correlations of trabecular parameters by μCT and bone histomorphometry trended toward increasing as the voxel size decreased from 91 µm to 8 µm **(** **Table 2** **)**, but the correlations were low (data not shown). Ten weeks after inoculation, a significant difference in all trabecular parameters except TbTh was seen between left and right femora on in vivo μCT and ex vivo μCT at all voxel sizes using optimal thresholding, concordant with histomorphometry. There were decreases in TbN and BV/TV, and increase in TbSp in femora with tumors compared to control femora by in vivo μCT or ex vivo μCT at all voxel sizes **(** **Figure 6B, C and D** **insets,** **Table 3** **)** using optimal thresholding, consistent with overall trabecular loss. This was not seen with automatic thresholding for TbN and BV/TV by in vivo μCT or ex vivo at 31 µm or with TbSp by in vivo or ex vivo μCT at any voxel size; this result demonstrates the importance of using fixed optimal thresholding instead of automatic thresholding. Fixed optimal thresholding demonstrated that prostate cancer involving bone altered trabecular morphology.

## Discussion

Prostate cancer involving the skeleton results in a host bone response that can be quantified by in vivo and ex vivo μCT in a mouse model. Although there was heterogeneity in the trabeculae affected by cancer (with thinning, thickening, and loss), overall there was trabecular loss in femora with tumor as exemplified by decreased TbN, BV/TV, and BMD, as well as increased TbSp; in contrast, diaphyseal cortex thickened. In addition to the effect of growth factors, data with the current mouse model suggest that a likely mechanism for the findings is tumor-induced loss of trabecular bone that causes both a trabecular bone reaction and cortical thickening to stabilize the loss of mechanical strength of the bone. This may be further tested in future studies. Findings also suggest that it is important to assess both cortical bone and trabecular bone since these may be discordant, especially in cancer. This suggests that it will become important to evaluate trabecular parameters as spatial resolution of clinical CT improves to a degree that allows such assessment.

We compared the abilities of three imaging methods to evaluate bone response to tumor: in vivo μCT, ex vivo specimen μCT and ex vivo DXA. All three imaging methods were able to distinguish BMD increases in the diaphyses, consisting primarily of cortical bone, of the femora with tumors. Compared to μCT-derived BMD, DXA-derived BMD had a lower coefficient of correlation with bone histomorphometry. This is consistent with findings by Barou et al. who also noted lower correlation between DXA and bone histomorphometry in a rat model [23]. In addition, DXA was unable to separate cortical bone from trabecular bone, whereas μCT could. A limitation of ex vivo μCT at the 8-µm voxel size was its small field of view that resulted in evaluation of only the metaphysis. It also required a relatively longer computing time. Ex vivo μCT at the 31 or 16-µm voxel size allowed imaging of the entire femur. With increasing resolution, we noted a trend toward higher correlation of trabecular parameters with histomorphometry, but the trend was not statistically significant. Surprisingly, even with relatively low spatial resolution, trabecular parameters could be assessed by in vivo μCT, thus, enabling longitudinal assessment. This suggests that in vivo μCT may be used to evaluate change in mineralized bone density and trabecular architecture over time, which may be caused by disease or therapy, for example, in animal models. In vivo μCT and ex vivo μCT quantified the BMD of the cortex and trabeculae well. In comparing different imaging modalities/machines for assessing BMD, it is commonly necessary to add a correction factor [30]. BMD determined by ex vivo μCT was 2.2 times higher than the BMD determined by in vivo μCT and because the correlation between these modalities was high, a correction factor of 2.2 could be applied when comparing in vivo μCT BMD with ex vivo μCT BMD.

In vivo or ex vivo μCT trabecular morphometric parameters (TbN, TbSp, and BV/TV) correlated well with bone histomorphometry. Most μCT studies of bones have used samples from rats [21], [23], [31] or humans [32]-[34], who have larger bones and trabeculae than mice. Owing to differences in size among species, voxel size should be considered. Muller et al. [35] found high correlations for BV/TV (r = 0.91) and TbSp (r = 0.91) of human bone biopsy specimens by 14-µm 3D μCT and histomorphometry. For BV/TV, TbN, and TbTh of human ilium, Uchiyama, et al. [36] reported correlations of r = 0.95, 0.75, 0.86, respectively, comparing 2D μCT (spatial resolution, 26-µm) and bone histomorphometry. For BV/TV, TbN, TbSp, and TbTh of human vertebrae, Peyrin et al. noted high correlation (r>0.93) between 6.6 µm Synchrotron CT and histomorphometry [33]. We also found that TbTh derived from in vivo μCT moderately correlated with ex vivo μCT at all voxel sizes; however, poor correlation was observed between TbTh by bone histomorphometry and by in vivo or ex vivo μCT. This may be due to several factors including volume averaging of the small mouse trabeculae [37], [38] and sampling error, since only three bone slices (right, mid, and left sagittal planes/fermur) per femur were assessed by bone histomorphometry and bone loss in the metaphysis was expectedly inhomogeneous in the tumor model. Correlations of TbTh were higher among μCT methods. Using a rat model of disuse osteoporosis, Barou, et al. [23] found poor correlation for TbTh by μCT versus bone histomorphometry and suggested that 3D- μCT may be more sensitive than histomorphometry in detecting changes in BMD and trabeculae.

In contrast to assessment of BMD, where thresholding was not a factor, the choice of thresholding significantly affected the assessment of trabecular morphometric parameters. Automatic thresholding and maximum gray-scale thresholding led to variability in morphometric values between animals and the femora with and without tumors. Further, neither correlated well with trabecular parameters as assessed by histomorphometry. In comparison, correlations of morphometric trabecular parameters (TbN, BV/TV, and TbSp) by histomorphometry with μCT were high using a fixed “optimal threshold”. We found that μCT at each voxel size had a separate “optimal” fixed threshold. To our knowledge, these findings have not been reported previously. Supporting our data, Ruegsegger et al. [32], [37] showed in a human iliac crest biopsy specimen model that a 10% change in the threshold resulted in a 5% change in BV/TV using 28 µm CT. Bouxsein et al. [39] described trabecular and cortical bone changes of inbred strains of mice using μCT with a threshold of 22% of maximal gray-scale value for vertebrae and tibia and 30% for mid-femoral cortical bone. In the current setting, we found that maximal gray scale values can vary and may not overlap with “optimal thresholds,” especially when tumor is present. Using automatic thresholding, differences between morphologic trabecular parameters were difficult to distinguish between femora with and without tumor; whereas, such differences were clearly appreciated using “optimal” fixed thresholds and by bone histomorphometry. Therefore, our data suggests that using fixed optimal thresholds is important for obtaining robust trabecular morphometric parameter values.

The results of this study using μCT to quantitatively characterize the mineralized component of mouse femora may be applicable to other studies of bones in small animals and humans. For example, one may evaluate bone response to strategies aimed at prevention or treatment of prostate bone metastases, primary bone tumor, or metastases from other primary tumors. The results also have the potential to be generalized to other bone models, such as metabolic bone disease.

Clinical multi-slice CT scanners are approaching resolutions fine enough for assessing trabeculae. Our data imply that each voxel size used will require optimization of thresholding. Automatic thresholding or maximum gray-scale thresholding will need validation because they may be too variable to achieve consistent results clinically. A fixed optimal threshold may be superior for assessing morphometric parameters. Because 70% of the effects of metabolic bone disease are first reflected in the trabeculae and only 30% in the cortex [40] and because bone metastases begin in the marrow, evaluation of the trabeculae may detect metabolic bone disease and we suspect also metastatic disease earlier than evaluation of the cortex. Since BMD was independent of voxel size and thresholding in the current study and given the sub-millimeter voxel size of new clinical scanners, it is feasible that clinical multi-slice CT may soon be used to determine BMD of the trabeculae to assess metabolic and neoplastic disease in patients. This requires further study. Morphometric parameters may also be added as clinical scanner voxel size improves. Findings suggest that separately evaluating cortex and trabeculae is important because the effects of pathology on the mineralized components of these two types of bone may be discordant.

In vivo μCT enables noninvasive, longitudinal assessment of mineralized bone. Ex vivo specimen μCT enables nondestructive assessment of mineralized bone enabling further study, for example, by histology. With appropriate thresholding, in vivo and ex vivo μCT can be used to quantify host response of both cortical and trabecular mineralized bone to prostate cancer involving the skeleton, and such a response may be different between these two mineralized bone compartments.
